# Integrative Single- and Multi-Trait GWASs Identify Pleiotropic Loci Affecting Growth and Egg Production in Zhedong Geese

**DOI:** 10.3390/ani16071072

**Published:** 2026-04-01

**Authors:** Wei Zhou, Jianhong Pan, Shiheng Zhou, Jingjing Yang, Linfang Wang, Pan Li, Chunyuan Zhang, Zhihao Jiang, Panxue Wu, Jindong Ren, Rongyang Li, Lizhi Lu, Li Chen, Zhenyang Zhang

**Affiliations:** 1 Xianghu Laboratory, Qianwan Bioport, 3300 Benjing Avenu, Xiaoshan District, Hangzhou 311231, China; 2 Zhejiang-Ukraine Joint Laboratory for Poultry Germplasm Resources Conservation, Exploitation and Utilization, Institute of Animal Science and Veterinary, Zhejiang Academy of Agricultural Sciences, Hangzhou 310021, China

**Keywords:** growth, egg, multi-trait GWAS, pleiotropic

## Abstract

Growth and egg production are the two most economically important traits in goose production systems. However, negative genetic correlations between these traits make it difficult to achieve balanced genetic improvement through selection. In this study, we analyzed whole-genome resequencing data from 1033 Zhedong White Geese to identify genetic variants related to birth weight (BW), body weight at 90 days (BW90), and egg number at 66 weeks of age (EN66). Single-trait genome-wide association studies (GWASs) identified 6, 5, and 5 lead SNPs significantly associated with BW, BW90, and EN66, respectively. By integrating network analysis, PLACO, and multivariate linear mixed models (mvLMMs), we further identified *KCNAB2* and *KCND3* as potential pleiotropic candidate genes influencing both growth and egg production. Notably, the variant at CHR25: 6006715, located within an intronic region of *KCND3*, was associated with increased BW (ZscoreBW = 4.44) and decreased EN66 (ZscoreEN66 = −3.55), showing strong pleiotropic significance (P_PLACO = 4.88 × 10^−8^). Collectively, these findings provide new insights into the genetic architecture underlying the antagonistic relationship between growth and egg production in geese and offer valuable genetic targets for developing breeding strategies that jointly optimize growth performance and reproductive efficiency.

## Simple Summary

Improving both growth and egg production is a major goal in goose breeding, but these traits are often negatively related, making it difficult to enhance them at the same time. In this study, we analyzed genetic data from more than one thousand Zhedong White Geese to identify genes that influence body weight and egg production. We found several genetic markers associated with growth at birth and later stages, as well as egg number. Importantly, we identified specific genes that affect both traits simultaneously, helping to explain why faster growth is often linked to lower egg production. One key genetic variant was found to increase body weight while reducing egg number, highlighting the biological basis of this trade-off. These findings improve our understanding of how growth and reproduction are genetically connected in geese. This knowledge can help breeders develop more effective strategies to improve meat production while maintaining sufficient reproductive performance, ultimately supporting more efficient and sustainable poultry production.

## 1. Introduction

Growth and egg production are the two most economically important trait categories in goose production systems. However, in geese, rapid growth is often associated with relatively low egg production, thereby limiting reproductive efficiency. Growth-related traits, such as birth weight, body weight at different developmental stages, and growth rate, directly determine market value in geese. Previous studies have identified numerous genomic regions and candidate genes associated with growth performance, including loci involved in muscle development [1] and energy metabolism [2] in the poultry species. In parallel, egg production traits, such as age at first egg, total egg number, and laying persistency, are critical determinants of reproductive efficiency in breeding populations. Genetic studies on egg production have highlighted the complex polygenic architecture of reproductive traits, primarily involving the hypothalamic–pituitary–ovarian axis [3,4].

Growth and reproductive traits are often genetically correlated. For example, in White Leghorn chickens, highly negative genetic correlations were observed between egg weight at 40 weeks, body weight at 52 weeks, and later egg production traits [5]. In Thai Native Synthetic chickens, the genetic correlations between body weight and egg production traits ranged from −0.48 to 0.15 [6]. Such antagonistic relationships indicate that selection for rapid growth may compromise egg number, emphasizing the need to identify genetic variants influencing both traits. These findings motivate the use of genomic approaches, such as genome-wide association studies (GWASs), to uncover loci with pleiotropic effects. For example, *MAN2A2* was strongly associated with average egg-laying interval in Jilin White Geese identified by GWASs [7]. Multi-trait GWASs further enable the detection of pleiotropic loci that simultaneously affect growth and reproductive performance, providing opportunities to mitigate antagonistic genetic correlations through informed selection strategies [8]. In female broilers, three candidate genes (*CPEB3*, *MAST2*, and *CACNA1H*) were linked to antagonistic pleiotropy, while *ACVR1* exhibited synergistic pleiotropic effects between body weight (BW) and egg number (EN) traits by bivariate association analyses [9].

Therefore, in this study, we used comprehensive genotype and phenotype data from the Zhedong White Goose population and applied both single-trait and multi-trait GWAS approaches to identify pleiotropic loci and candidate genes underlying growth and egg production traits. By integrating multiple analytical strategies, we aimed to uncover shared genetic signals affecting body weight and egg number, thereby providing a genetic foundation for the coordinated improvement in growth and reproductive performance in goose breeding programs.

## 2. Methods

### 2.1. Ethical Statement

All animal experiments were approved by the Animal Ethics Committee of Zhejiang Academy of Agricultural Sciences (Approval Number: 2021ZAASLA32).

### 2.2. Study Population and Phenotype

All phenotypic data were collected from the same batch of 1033 purebred broiler Zhedong White geese. Body weight was recorded at birth and again at 90 days of age. Throughout the rearing period, all individuals were maintained under uniform housing conditions and standardized nutritional management to minimize environmental variation.

Subsequently, 796 female geese were transferred to individual laying cages for egg production recording. Egg numbers were continuously recorded for more than 66 weeks for each individual, and the total egg number at 66 weeks of age was used as the egg production trait for downstream analyses.

### 2.3. Genotype and Quality Control

For each individual, 2 mL of whole blood was collected from the wing vein using EDTA anticoagulant tubes (Jiangsu Kangjian Medical Apparatus Co., Ltd., Taizhou, China). Genomic DNA was extracted and subjected to whole-genome sequencing on the BGI platform (BGISEQ, DNBSEQ-T7, PE 150 model, MGI Tech Co., Ltd., Shenzhen, China). The average sequencing depth was 8.6×, and 96.5% of bases achieved a quality score of Q30 (Supplementary Table S1).

Raw sequencing reads were first quality-controlled using fastp (0.23.2) [10] to remove low-quality reads and adapter contamination. Duplicate reads were then identified and marked using Sambamba v1.0.1 [11]. Clean reads were aligned to the reference genome (GCF_040182565.1 [12]) using the BWA-MEM [13] algorithm, and genetic variants were called using the GATK (4.3.0.0) [14] pipeline following best-practice recommendations.

Variants were filtered based on a minor allele frequency (MAF) ≥ 0.05 and a call rate ≥ 0.9, resulting in 7,664,463 high-quality SNPs (Supplementary Figure S1). To estimate the number of approximately independent markers, linkage disequilibrium (LD) pruning was conducted using PLINK v1.9.0 [15] with the parameters “--indep-pairwise 50 5 0.2”, yielding 318,887 independent SNPs for downstream analyses.

### 2.4. Estimation of Genetic Parameters

Genetic parameters were estimated by BGLR R package [16]:

(1) y=Xb+Za+e

where y is the vector of phenotypic values, b is the corresponding vector of fixed-effect coefficients (sex), a represents the vector of additive genetic effects of the animal, with $a\sim N(0,K\sigma_{a}^{2})$. **K** is the kernel (similarity) matrix constructed from the genomic relationship matrix (**G**) and $\sigma_{a}^{2}$ is the additive genetic variance. **G** matrix was constructed as [17]. e is the vector of random residuals, with *N* (0, **I**$\sigma_{e}^{2}$), and **X** and **Z** are the corresponding incidence matrices.

The narrow-sense heritability (h^2^) was computed from the additive genetic variance and the total phenotypic variance via the following formula:

(2) $$h^{2}=\frac{\sigma_{a}^{2}}{\sigma_{a}^{2}+\sigma_{e}^{2}}$$

Model parameters were estimated using Markov Chain Monte Carlo (MCMC) sampling in BGLR, with specified 12,000 iterations and 2000 burn-in cycles. Posterior means of variance components were used to compute heritability and its standard error.

Genetic correlations were estimated through bivariate models between each pair of traits, as follows:

(3) ymyn=Xm00Xnbmbn+Zm00Znaman+emen

where y, X, b, Z, a, and e are identical to model (1). The subscripts m and n represent the mth and nth traits used for calculating genetic correlation. Where a **=** aman are assumed to follow normal distributions N(0, σa(m)2σa(m,n)σa(m,n)σa(n)2⨂K), e=emen are assumed to follow normal distributions N(0, σe(m)200σe(n)2⨂I). Where $\sigma_{a(m,n)}$ is the genetic covariance between m and n. $\sigma_{a(m)}^{2}$ and $\sigma_{a(n)}^{2}$ are the genetic variances of m and n, respectively. $\sigma_{e(m)}^{2}$ and $\sigma_{e(n)}^{2}$ are the residual variance of m and n, respectively; ⨂ is the Kronecker product.

The genetic correlations were calculated as follows:

(4) $$r_{g\left(m,n\right)}=\frac{\sigma_{a(m,n)}}{\sqrt{\sigma_{a(m)}^{2}\sigma_{a(n)}^{2}}}$$

### 2.5. Single-Trait and Multi-Trait GWASs

Population structure was first evaluated by principal component analysis (PCA) using PLINK v1.9.0 [15]. The PCA results indicated a genetically homogeneous population with no obvious population stratification (Supplementary Figure S2, Supplementary Table S2). The top three principal components (PCs) were included as covariates in the genome-wide association analyses. For birth weight and body weight at 90 days of age, sex was additionally fitted as a fixed effect.

Single-trait GWAS was conducted using the fastGWA [18] module implemented in GCTA [17] (v1.94.0). The model was:

(5) y=Xβ+Zα+ξ+e

where y is the vector of phenotypic values, X is the design matrix of fixed effects, and β is the corresponding vector of fixed-effect coefficients, including sex and the top three principal components to account for population structure. Z is the vector of SNP genotypes, coded as 0, 1, and 2 for genotypes A_1_A_1_, A_1_A_2_ and A_2_A_2_, respectively; α represents the additive genetic effect of the tested SNP, and the model was fitted separately for each SNP.

The random polygenic effect ξ follows a multivariate normal distribution, ξ ~ N (0, **G**$\sigma_{g}^{2}$), where **G** is the genomic relationship matrix (GRM) and $\sigma_{g}^{2}$ is the additive genetic variance. The residual error term e follows e ~ N (0, **I**$\sigma_{e}^{2}$), where ***I*** is the identity matrix and $\sigma_{e}^{2}$ is the residual variance. Variance components $\sigma_{g}^{2}$ and $\sigma_{e}^{2}$ were estimated using the grid-search-based restricted maximum likelihood (REML) algorithm.

The **G** matrix was constructed as follows [19]:

(6) $$G=MDM^{\prime }$$

where M is the SNP markers’ incidence matrix, elements of which are coded as 0-2$p_{i}$, 1-2$p_{i}$, 2-2$p_{i}$ for A_1_A_1_, A_1_A_2_ and A_2_A_2_ genotypes, respectively, and $p_{i}$ is the frequency of A_2_ allele for the *i*th locus. D is a diagonal matrix with $D_{ii}=\frac{1}{m[2p_{i}(1-p_{i})]}$, where m is the number of SNP.

To jointly analyze multiple correlated traits, a multivariate GWAS was conducted using the multivariate linear mixed model implemented in GEMMA v0.98.5 [8,20]. In this framework, the phenotype matrix y has dimensions n × d, where n denotes the number of individuals and d the number of traits. Fixed effects were modeled using the design matrix X (n × p), including sex and the top three principal components, with corresponding regression coefficients β (p × d), allowing each fixed effect to have trait-specific effects. For each SNP, the genotype vector Z (n × 1) was fitted with a vector of SNP effects α (1 × d), representing the additive genetic effects of the tested marker on each trait. Random polygenic effects were modeled as ξ ~ N (0, **G** ⊗ $V_{g}$), where $V_{g}$ (d × d) is the genetic variance–covariance matrix capturing shared genetic architecture among traits. Residual effects were modeled as e ~ N (0, **I**⊗ $V_{e}$), where $V_{e}$ represents the residual variance–covariance matrix.

The genome-wide significance threshold was determined using a Bonferroni correction (*p* < 0.05 divided by the number of independent SNPs), while a suggestive significance threshold was defined as *p* < 1 divided by the number of independent SNPs. Manhattan plots and QQ plots were generated via the CMplot v4.5.1 [21] function in the R package.

### 2.6. Pleiotropic Analysis Under Composite Null Hypothesis

To identify genetic variants with pleiotropic effects across traits, pleiotropic analysis was performed using the PLACO+ (Pleiotropic Analysis under Composite Null Hypothesis) method implemented in the PLACO v0.2.0 R package [22]. PLACO+ evaluates whether a given SNP is simultaneously associated with two traits by testing against a composite null hypothesis that allows the SNP to be associated with neither trait or only one of the traits, thereby effectively distinguishing true pleiotropic signals from trait-specific associations.

Single-trait GWAS summary statistics were used as input for the analysis. Prior to PLACO+ testing, allele harmonization was performed to ensure consistency of effect alleles and effect directions across traits. Ambiguous strand SNPs were removed to avoid potential alignment errors. For each SNP, Z-scores were calculated from the GWAS summary statistics and supplied to the PLACO+ algorithm. Statistical significance was assessed using PLACO-derived *p* values, with multiple testing correction applied using the Bonferroni method based on the number of independent SNPs. SNPs surpassing the corrected significance threshold were considered to exhibit significant pleiotropic effects between the analyzed traits.

### 2.7. Functional Annotation and Gene Network Analysis

Genetic variants were functionally annotated using ANNOVAR (version 20220822) [23] based on GCF_040182565.1. Annotated genes were subsequently subjected to functional enrichment analysis using KOBAS 3.0 [24].

To further explore functional relationships among candidate genes, gene–gene interaction networks were constructed using GeneMANIA (https://genemania.org/) prediction server [25]. In addition, protein–protein interaction (PPI) analysis was performed using the STRING database [26].

## 3. Results

### 3.1. Basic Statistics and Genetic Parameters

**Figure 1:**
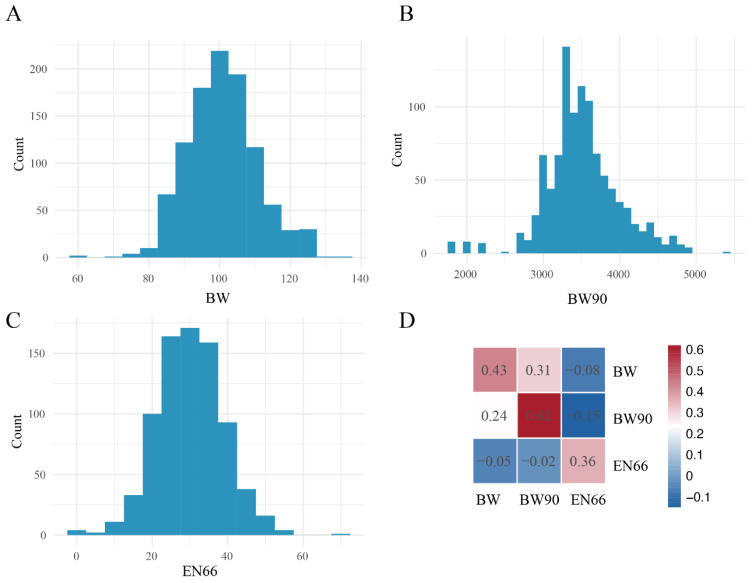
Phenotypic distributions and genetic parameter estimate of growth and egg production traits. (**A**) Phenotypic distribution of birth weight (BW); (**B**) Phenotypic distribution of body weight at 90 days of age (BW90); (**C**) Phenotypic distribution of egg number at 66 weeks of age (EN66); (**D**) Genetic parameter estimates, where the lower triangle represents phenotypic correlations, the upper triangle represents genetic correlations, and the diagonal elements indicate heritability estimates.

Egg production and growth traits are the most economically important phenotypes in goose production. In this study, birth weight (BW) ranged from 58.89 to 134 g, with a mean of 100.81 ± 9.95 g (Figure 1A). Body weight at 90 days of age (BW90) ranged from 1771 to 5390 g, with a mean of 3512.13 ± 485.64 g (Figure 1B). The egg number at 66 weeks of age (EN66) varied from 0 to 69, with an average of 30.05 ± 8.68 (Figure 1C).

Using a single-trait model, the heritability estimates (±standard error; Figure 1D) for BW, BW90, and EN66 were 0.43 ± 0.11, 0.62 ± 0.07, and 0.36 ± 0.11, respectively. A moderate positive genetic correlation (Figure 1D) was observed between BW and BW90 (r = 0.31 ± 0.14). In contrast, EN66 showed negative genetic correlations with growth traits. Specifically, the genetic correlation between EN66 and BW was −0.08 ± 0.18, a stronger negative correlation was observed between EN66 and BW90 (−0.15 ± 0.17). However, the standard errors of the genetic correlation estimates were relatively large, indicating considerable uncertainty in these estimates. To validate the robustness of these estimates, pairwise genetic correlations were independently calculated using GCTA, yielding a consistent estimate for EN66 and BW90 (−0.96 ± 0.86). In addition, estimated breeding values (EBVs) for individuals exhibited pronounced correlations among traits, supporting the observed genetic relationships (Supplementary Figure S3).

### 3.2. Single-Trait Genome-Wide Association Analyses of Growth and Egg Production Traits

**Figure 2:**
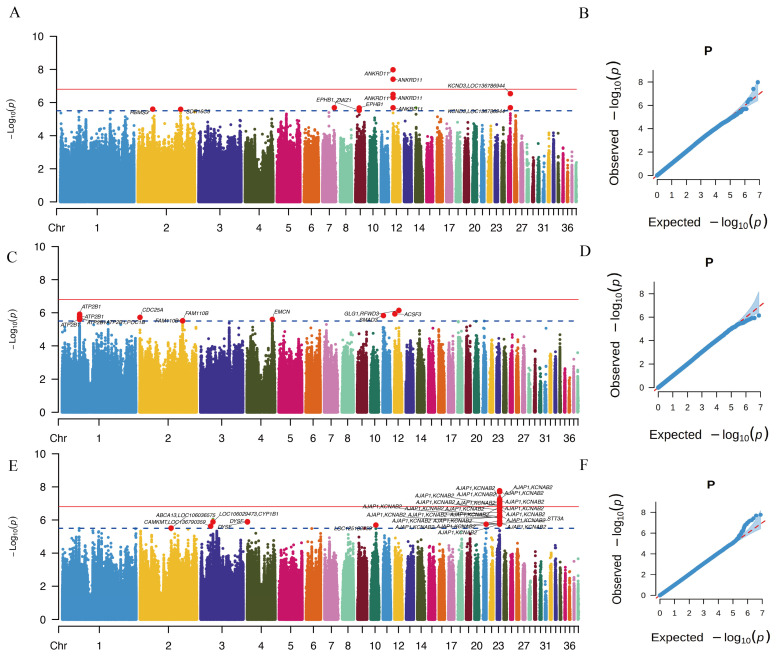
Single-trait GWAS results. (**A**), (**C**), and (**E**) show the Manhattan plots for birth weight (BW), 90-day body weight (BW90), and egg number at 66 weeks (EN66), respectively. (**B**), (**D**), and (**F**) present the corresponding QQ plots.

We performed a single-trait GWAS for BW (Figure 2A,B, Supplementary Table S3). The genomic inflation factor (λ) was 1.07, indicating minimal population stratification. Conditional and joint analysis (COJO) further identified five independent genome-wide significant loci and one indel associated with BW (Table 1). Gene annotation revealed that these variants were located in or near *ANKRD11*, *KCND3*, *LOC136786944*, *ZMIZ1*, *LOC106032756*, *SLC22A4*, *EPHB1*, and *RBMS3* (Table 1). The most significant SNP was CHR12:1,550,139 (β = −2.88, *P* = 1.04 × 10^−8^), which is located within an intron of *ANKRD11* gene. *ANKRD11* has been implicated in reduced neonatal body size and KBG syndrome in humans [27]. In addition, mutations in *KCND3* have been associated with cerebellar ataxia, intellectual disability, epilepsy, and attention deficit disorders [28]. Functional enrichment analysis showed that these genes were significantly enriched in GO terms related to vitellogenesis (*FDR* = 0.01), axon guidance receptor activity (*FDR* = 0.01), and so on (Supplementary Table S4).

For BW90, a total of five independent genome-wide significant loci were identified (Table 1, Figure 2C,D, Supplementary Table S3), with a genomic inflation factor of λ = 1.09. The candidate genes near these loci included *GLG1*, *RFWD3*, *ACSF3*, *ATP2B1*, *SMAD3*, and *CDC25A*. Notably, the SNP CHR11:2,038,674 was located within an intron of *SMAD3*. It is related to the TGF-beta signaling pathway and FoxO signaling pathway (*FDR* = 0.04) (Supplementary Table S5). *SMAD3* functions as a key transcription factor that, activated by the TGF-β signaling pathway, directly binds to the *IGFBP5* promoter to initiate granulosa cell degeneration, leading to follicular atresia in chickens [29].

For EN66, five independent genome-wide significant loci were identified (Table 1, Figure 2E,F, Supplementary Table S3), with a genomic inflation factor of λ = 1.09. The putative candidate genes included *AJAP1*, *KCNAB2*, *RMDN2*, *DYSF*, *STT3A*, and LOC125183753. *STT3A*, *RMDN2*, and *DYSF* were enriched in integral component of membrane GO term significantly (*FDR* = 0.017) (Supplementary Table S6). The most significant SNP (CHR23: 7,748,540) was located within an intron of *KCNAB2*, which encodes a β-subunit of voltage-gated potassium channels and modulates channel inactivation and electrical signaling in neuronal and endocrine cells. In addition, *RMDN2* has been reported to be differentially expressed in chicken granulosa cells during follicular development, and polymorphisms in this gene were significantly associated with egg number at 40 weeks of age in laying hens [30].

### 3.3. Gene Network Analysis and Pleiotropic Effects on Growth and Egg Production Traits

**Figure 3:**
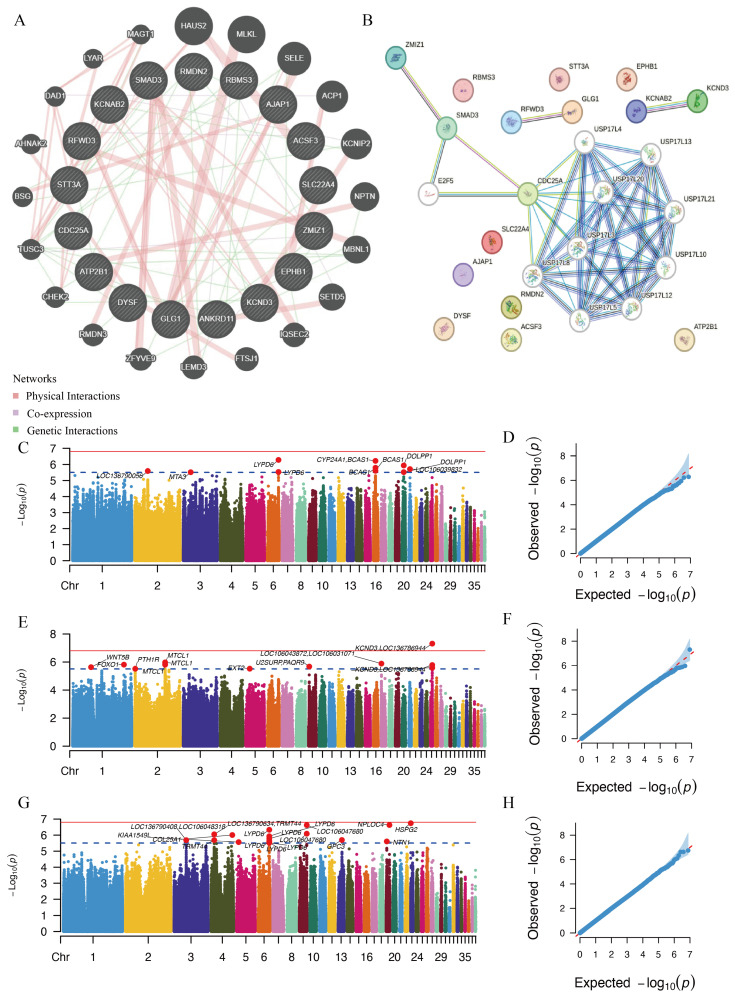
Candidate gene network analysis and PLACO pleiotropic analysis results. (**A**) Gene interaction network constructed using GeneMANIA. (**B**) Protein–protein interaction network derived from the STRING database. (**C**,**D**) Manhattan plot and corresponding QQ plot of PLACO results for BW and BW90. (**E**,**F**) Manhattan plot and corresponding QQ plot of PLACO results for BW and EN66. (**G**,**H**) Manhattan plot and corresponding QQ plot of PLACO results for BW90 and EN66.

To investigate potential relationships among candidate genes shared by the three traits, we examined the interactions among the 20 associated genes using the GeneMANIA and STRING databases. Extensive protein–protein interaction networks were observed among *ZMIZ1*, *SMAD3*, *E2F5*, and *CDC25A* (Figure 3A,B). Notably, *KCNAB2* and *KCND3* exhibited both physical interactions and genetic interactions in the GeneMANIA network (Figure 3A). Consistently, the STRING database also indicated multiple lines of functional association between *KCNAB2* and *KCND3* (Figure 3B).

These results suggest that candidate genes underlying different traits are functionally interconnected. Therefore, we further conducted a pleiotropic analysis using PLACO under the composite null hypothesis. For BW and BW90, a total of 10 genetic variants reached the suggestive significance threshold (Figure 3C,D; Supplementary Table S7), located near *LYPD6*, *CYP24A1*, *BCAS1*, *DOLPP1*, LOC106039832, LOC136790056, and *MTA3*.

Between BW and EN66, 12 genetic variants exceeded the suggestive threshold (Figure 3E,F; Supplementary Table S8), mapping to regions near *KCND3*, LOC136786944, *MTCL1*, LOC106043872, LOC106031071, *FOXO1*, *U2SURP*, *PAQR9*, *WNT5B*, *EXT2*, and *PTH1R*. Among these, CHR25: 6006715 reached genome-wide significance (*p* = 4.88 × 10^−8^) and is located within an intronic region of *KCND3*.

For BW90 and EN66, 17 genetic variants showed suggestive pleiotropic signals (Figure 3G,H; Supplementary Table S9), distributed near *HSPG2*, *NPLOC4*, LOC106047680, *LYPD6*, LOC136790634, *TRMT44*, *COL25A1*, *GPC3*, LOC136790408, LOC106048318, *NTN1*, and *KIAA1549L*.

### 3.4. Identification of Pleiotropic Loci by Multivariate Linear Mixed Models

**Figure 4:**
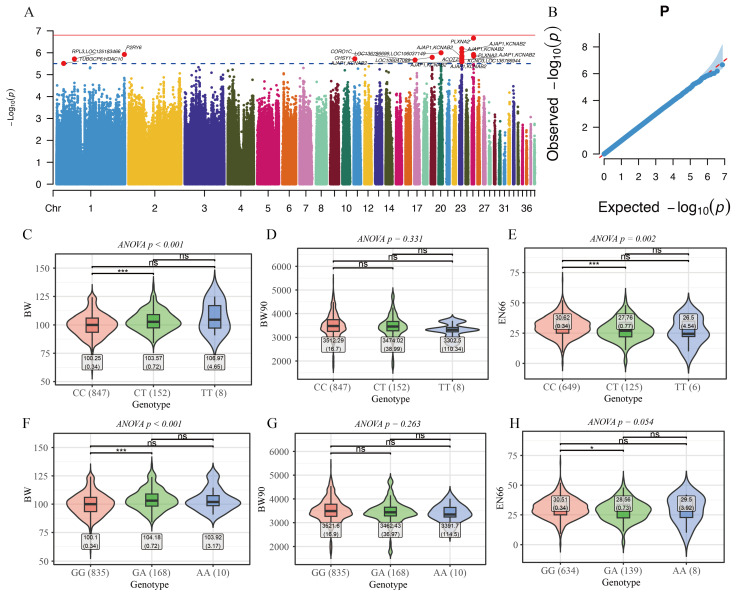
mvLMM results and phenotypic variation at pleiotropic loci. (**A**) show the Manhattan plot from the mvLMM analysis (**B**) presents the corresponding QQ plot. (**C**–**E**) are boxplots showing birth weight (BW), 90-day body weight (BW90), and egg number at 66 weeks (EN66), respectively, across genotypes at CHR25: 6006715. (**F**–**H**) are boxplots showing BW, BW90, and EN66, respectively, across genotypes at CHR25: 6004533. The *x*-axis indicates genotypes, with sample sizes shown in parentheses, and the *y*-axis represents the phenotype value for the corresponding traits. Gray boxes denote mean values with standard errors (SE). Statistical significance based on *t*-tests is indicated above the boxplots. * *p* < 0.05; *** *p* < 0.001; ns, not significant.

To further identify pleiotropic genetic variants affecting all three traits, we applied a multivariate linear mixed model (mvLMM) to detect pleiotropic loci. In total, 18 variants exceeded the suggestive significance threshold (λ = 1.1) (Figure 4A,B; Supplementary Table S10). These loci were annotated to genes including *PLXNA2*, *AJAP1*, LOC136786699, LOC106037149, *P2RY6*, *ACOT7*, LOC106047089, *CHSY1*, *RPL3*, LOC125183466, *CORO1C*, *TUBGCP6*, and *HDAC10*. *PLXNA2* encodes Plexin A2, a semaphorin receptor involved in axon guidance, cell migration, and neural development. In mammals, it has been linked to brain development and neurodevelopmental phenotypes [31]. *CHSY1* encodes chondroitin sulfate synthase 1, involved in glycosaminoglycan biosynthesis, important for cartilage and extracellular matrix development. Interestingly, a pleiotropic GWAS in broilers identified *PLXNA2* and *CHSY1* as part of a shared gene set related to concurrent growth/egg number traits, indicating these two genes may participate in pathways that affect both body growth and reproductive processes through underlying biological systems such as morphogenesis and development [9].

Furthermore, mvLMM and PLACO independently identified CHR25: 6004533 and CHR25: 6006715 as pleiotropic loci jointly associated with body weight and egg production. Both variants are located within intronic regions of *KCND3*. The most prominent mvLMM signal was observed at CHR23: 7744628-7752457, primarily spanning intronic regions of *KCNAB2*. Together, these results implicate *KCND3* and *KCNAB2* as pleiotropic genes influencing both growth and egg production.

By further examining the relationship between genotypes at the pleiotropic locus (CHR25: 6006715 and CHR25: 6004533) and individual phenotypes (Figure 4C–H) as well as estimated breeding values (Supplementary Figure S4), we found that the AB genotype was associated with a significantly increased BW (Figure 4C,F) but a reduced EN66 (Figure 4E,H). Interestingly, no significant association was observed between the three genotypes and BW90 (Figure 4D,G). This pattern is potentially favorable from a breeding perspective, as it suggests that despite the significant genetic correlation between BW90 and EN66, there exist variants that primarily affect EN66. Under such circumstances, selecting the AA genotype could maintain BW at an optimal level without altering BW90, while increasing egg production by approximately 2–3 eggs. However, the GWAS power for BW90 in this study was relatively limited, as indicated by the QQ plot (Figure 2D), and whether the AB genotype influences BW90 requires further validation using larger datasets and experimental evidence.

## 4. Discussion

Egg production is well known to be influenced by multiple genetic and environmental factors, including age at sexual maturity, nutrition, photoperiod, and breed. In the present study, the heritability of egg number in the Zhedong White goose population was relatively low (0.09 ± 0.17), which is lower than that reported for Zatorska geese (0.12–0.24) [32]. However, the latter estimates were calculated based on average egg numbers within specific laying periods. For comparable traits, Chen et al. [33] reported a heritability of 0.08 ± 0.01 for egg number in ducks between 18 and 58 weeks of age, which is consistent with our results.

For BW90, the heritability estimated using a single-trait model was low (0.04 ± 0.13), whereas the estimate increased substantially under the multi-trait model (0.21 ± 0.13). This improvement is likely attributable to the relatively strong genetic correlations among traits, allowing for shared genetic information to be leveraged. Consistently, Yang et al. [34] reported that incorporating growth traits can improve the prediction accuracy of reproductive traits. These results suggest that multi-trait models may be useful for improving EBV prediction accuracy in geese.

The primary objective of this study was to identify pleiotropic loci underlying body weight and egg production. Single-trait GWAS revealed that genetic variants near *KCND3* and *KCNAB2* were significantly associated with BW and EN66, respectively. Network analyses further suggested extensive functional connections between these two genes. In addition, *KCND3* exhibited pleiotropic effects on BW and EN66 in the PLACO analysis, and both *KCND3* and *KCNAB2* showed significant signals in the mvLMM analysis. Collectively, these lines of evidence indicate that *KCND3* and *KCNAB2* are likely involved in the regulation of both birth weight and egg production in geese. However, no goose-specific functional evidence is currently available to confirm their biological roles in these traits. The interpretation of these genes is therefore based primarily on conserved ortholog and previously reported functions in other species. *KCNAB2* encodes a voltage-gated potassium channel β-subunit that modulates channel gating properties by regulating inactivation kinetics and membrane trafficking of Kv1 channel complexes [35]. It was reported to participate in the regulation of growth hormone signaling, linking potassium channel activity to somatic growth and endocrine control [36,37]. The *KCND3* encodes the Kv4.3 α-subunit, which play a critical role in action potential repolarization for neurons and cardiac myocytes [38,39]. Kv channels regulate energy homeostasis [40], body weight [41] and hypothalamus–pituitary–ovarian axis [42]. This study suggests that variation in *KCNAB2* and *KCND3* may affect both egg production and growth. These findings, together with previous reports, indicate that these genes may exert their effects through the regulation of potassium ion channels. However, further experimental validation, including gene expression profiling and functional assays, will be required to clarify whether and how variation in these loci contributes to growth and reproductive traits in geese.

Despite the multiple lines of evidence supporting the identification of pleiotropic genes in this study, several limitations should be acknowledged. First, the relatively large standard errors observed for heritability and genetic correlation estimates are likely attributable to the limited sample size and the inherent sampling variance associated with variance component estimation. Genetic correlation estimates are especially sensitive to sample size and trait heritability, and thus tend to exhibit greater uncertainty when traits have low-to-moderate heritability. Future studies incorporating additional batches and larger sample sizes will be necessary to improve the stability and reliability of these estimates. Second, the present study was conducted in a commercial purebred breeding population. While this design reflects practical breeding conditions, the genetic variability within such a population may be limited compared to crossbred populations. The use of experimental F2 populations derived from divergent parental lines (e.g., broiler × layer crosses) could generate greater genetic recombination and phenotypic variation, thereby increasing mapping resolution and facilitating the identification of causal variants underlying growth and reproductive traits. Such designs may provide complementary advantages for fine-mapping pleiotropic loci in future studies. Third, the present study included only two growth-related traits and one egg production trait. Future research should incorporate repeated measurements of body weight across multiple developmental stages and model both growth and egg production as longitudinal traits to better capture dynamic genetic effects.

## 5. Conclusions

In this study, single-trait GWAS identified multiple loci independently associated with BW, BW90, and EN66, while integrative PLACO and multi-trait GWAS analyses consistently highlighted *KCND3* and *KCNAB2* as pleiotropic genes affecting both growth and egg production in geese. These results demonstrate that antagonistic relationships between body weight and egg number can be partially explained by shared genetic variants with pleiotropic effects. The identification of such loci provides practical opportunities to incorporate multi-trait and marker-assisted selection strategies aimed at improving egg production without severely compromising growth performance. Collectively, our findings offer valuable genomic resources and biological targets for optimizing balanced breeding schemes and enhancing overall reproductive efficiency in goose breeding programs.

## Figures and Tables

**Table 1:** Independent genome-wide significant loci identified by conditional and joint (COJO) analysis in single-trait GWAS. The “Trait” column indicates the phenotypes with which each significant variant is associated (BW, BW90, or EN66).

Trait	Chr	SNP	bp	refA	freq	b	se	p	n	freq_geno	bJ	bJ_se	pJ	Gene	Func.refGene
BW	12	12: 1550139	1550139	A	0.22	−2.88	0.5	1.04 × 10^−8^	1015.26	0.22	−2.88	0.51	1.74 × 10^−8^	*ANKRD11*	intronic
BW	25	25: 6004533	6004533	A	0.09	3.8	0.74	2.91 × 10^−7^	957.26	0.09	3.8	0.75	4.13 × 10^−7^	*KCND3*, *LOC136786944*	intronic
BW	7	7: 34215082	34215082	C	0.16	−2.66	0.56	2.05 × 10^−6^	1049.23	0.16	−2.66	0.57	2.59 × 10^−6^	*ZMIZ1*	intronic
BW	14	14: 1909343	1909343	G	0.22	−2.42	0.51	2.05 × 10^−6^	1011.21	0.22	−2.42	0.52	2.62 × 10^−6^	*LOC106032756*, *SLC22A4*	Indel
BW	9	9: 10670358	10670358	T	0.1	3.24	0.68	2.12 × 10^−6^	1042.74	0.1	3.24	0.69	2.69 × 10^−6^	*EPHB1*	intronic
BW	2	2: 42746129	42746129	G	0.07	−3.62	0.77	2.51 × 10^−6^	1089.15	0.07	−3.62	0.78	3.13 × 10^−6^	*RBMS3*	intronic
BW90	12	12: 13358999	13358999	G	0.13	−142.64	28.77	7.16 × 10^−7^	1025.6	0.13	−142.64	29.1	9.54 × 10^−7^	*GLG1*, *RFWD3*	intergenic
BW90	12	12: 1669403	1669403	T	0.07	−182.08	37.46	1.17 × 10^−6^	993.19	0.07	−182.08	37.89	1.54 × 10^−6^	*ACSF3*	intronic
BW90	1	1: 50540880	50540880	C	0.23	−113.93	23.48	1.22 × 10^−6^	975.11	0.23	−113.93	23.75	1.61 × 10^−6^	*ATP2B1*	intronic
BW90	11	11: 2038674	2038674	T	0.39	94.29	19.59	1.48 × 10^−6^	1056.72	0.39	94.29	19.79	1.90 × 10^−6^	*SMAD3*	intronic
BW90	2	2: 869036	869036	C	0.34	97.44	20.45	1.90 × 10^−6^	1026.11	0.34	97.44	20.67	2.43 × 10^−6^	*CDC25A*	intronic
EN66	23	23: 7748540	7748540	G	0.09	4.21	0.75	1.70 × 10^−8^	724.97	0.1	4.21	0.76	3.32 × 10^−8^	*AJAP1*, *KCNAB2*	intronic
EN66	3	3: 35535599	35535599	T	0.21	2.55	0.52	8.88 × 10^−7^	768.44	0.21	2.55	0.53	1.28 × 10^−6^	*RMDN2*	Indel
EN66	4	4: 810862	810862	A	0.06	−4.33	0.89	1.25 × 10^−6^	781.47	0.06	−4.33	0.91	1.76 × 10^−6^	*DYSF*	intronic
EN66	21	21: 9290060	9290060	G	0.38	−2.02	0.42	1.80 × 10^−6^	811.87	0.38	−2.02	0.43	2.46 × 10^−6^	*STT3A*	intronic
EN66	10	10: 14508858	14508858	G	0.1	3.4	0.72	2.03 × 10^−6^	766.18	0.1	3.4	0.73	2.79 × 10^−6^	*LOC125183753*	ncRNA_exonic

## Data Availability

The data presented in this study are not publicly available due to commercial restrictions. The data can be made available from the corresponding author upon reasonable request and with permission from the data provider.

## Supplementary Materials

The following supporting information can be downloaded at: https://www.mdpi.com/article/10.3390/ani16071072/s1, Figure S1. Genome-wide distribution of genetic variant density; Figure S2. Principal component analysis (PCA) based on genome-wide SNP data; Figure S3. Scatter plots showing correlations among estimated breeding values (EBVs) for different traits. Panel A shows the relationship between birth weight (BW; x-axis) and body weight at 90 days (BW90; y-axis). Panel B shows the relationship between birth weight (BW; x-axis) and egg number at 66 weeks (EN66; y-axis). Panel C shows the relationship between body weight at 90 days (BW90; x-axis) and egg number at 66 weeks (EN66; y-axis); Figure S4. Changes in EBVs across genotypes at pleiotropic loci. Panels A-C correspond to CHR25:6006715, and panels D-F correspond to CHR25:6004533. Panels A and D, B and E, and C and F show results for birth weight (BW), body weight at 90 days (BW90), and egg number at 66 weeks (EN66), respectively. The x-axis indicates genotypes (with sample sizes shown in parentheses), and the y-axis represents the EBV of the corresponding trait. Gray boxes denote the mean ± standard error (SE). Statistical significance based on *t*-tests is indicated above the boxplots. Table S1. Signicant variants detected in the GWAS; Table S2. Eigenvalues generated by PCA; Table S3. Signicant variants detected in the GWAS; Table S4. GO and KEGG enrichment analysis results for genes associated with birth weight (BW); Table S5. GO and KEGG enrichment analysis results for genes associated with body weight at 90 days (BW90); Table S6. GO and KEGG enrichment analysis results for genes associated with egg number at 66 weeks (EN66); Table S7. PLACO results for pleiotropic analysis between birth weight (BW) and body weight at 90 days (BW90); Table S8. PLACO results for pleiotropic analysis between birth weight (BW) and egg number at 66 weeks (EN66); Table S9. PLACO results for pleiotropic analysis between body weight at 90 days (BW90) and egg number at 66 weeks (EN66); Table S10. Results of the multivariate linear mixed model (mvLMM) analysis.
