# Supplementary material for: Integrative Single- and Multi-Trait GWASs Identify Pleiotropic Loci Affecting Growth and Egg Production in Zhedong Geese
Source: Animals (Basel). 2026 Apr 1;16(7):1072. doi: 10.3390/ani16071072 (PMC13072256; doi:10.3390/ani16071072)
Supplement: Supplementary file 1 [file animals-16-01072-s001.zip › Supplementary Files/Supplementary Figures.docx]

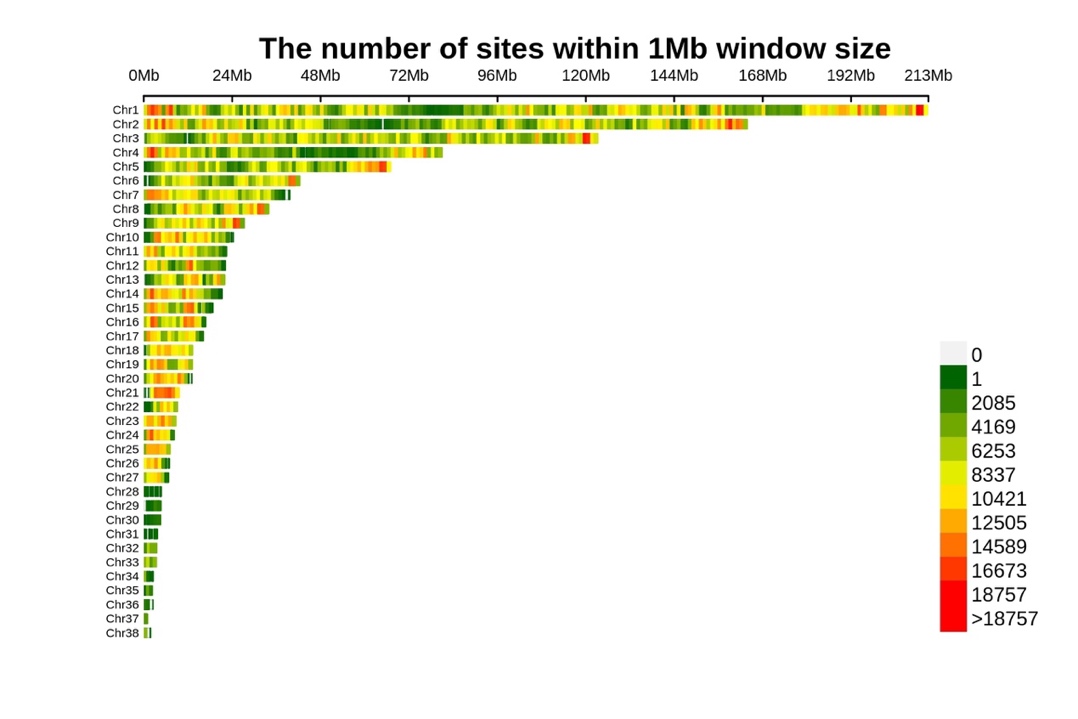


Supplementary Figure S1. Genome-wide distribution of genetic variant density.


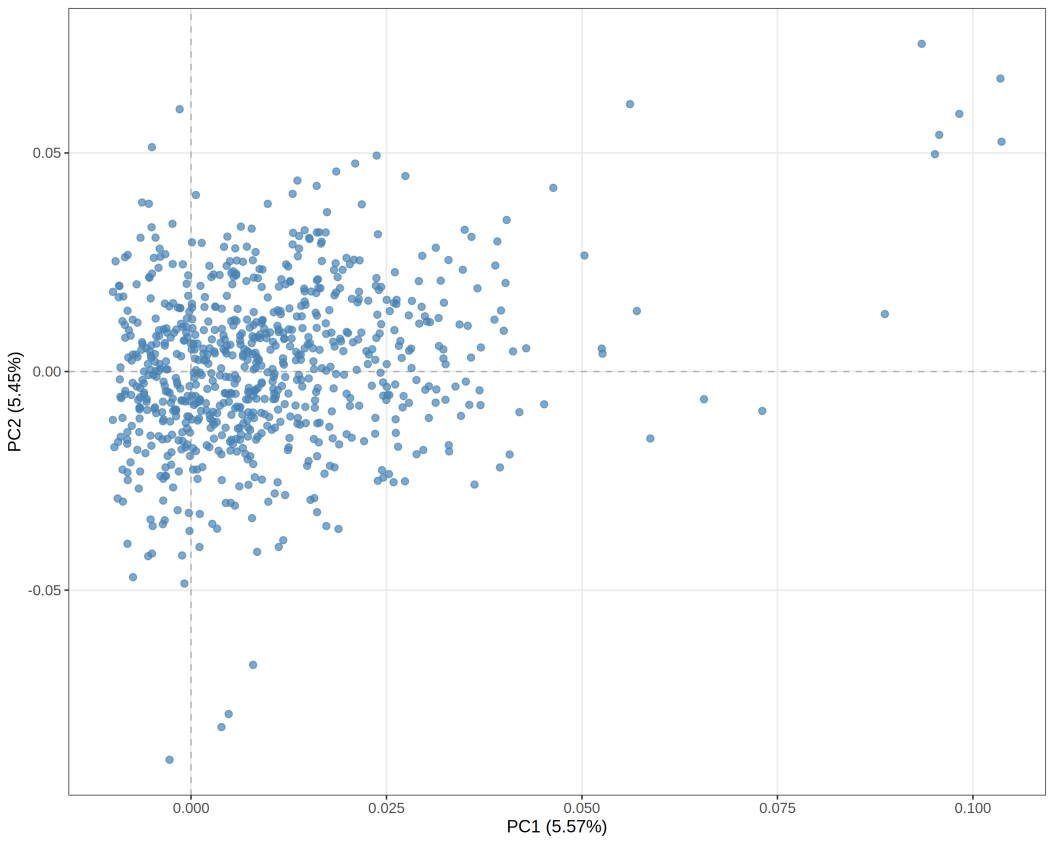


Supplementary Figure S2. Principal component analysis (PCA) based on genome-wide SNP data.


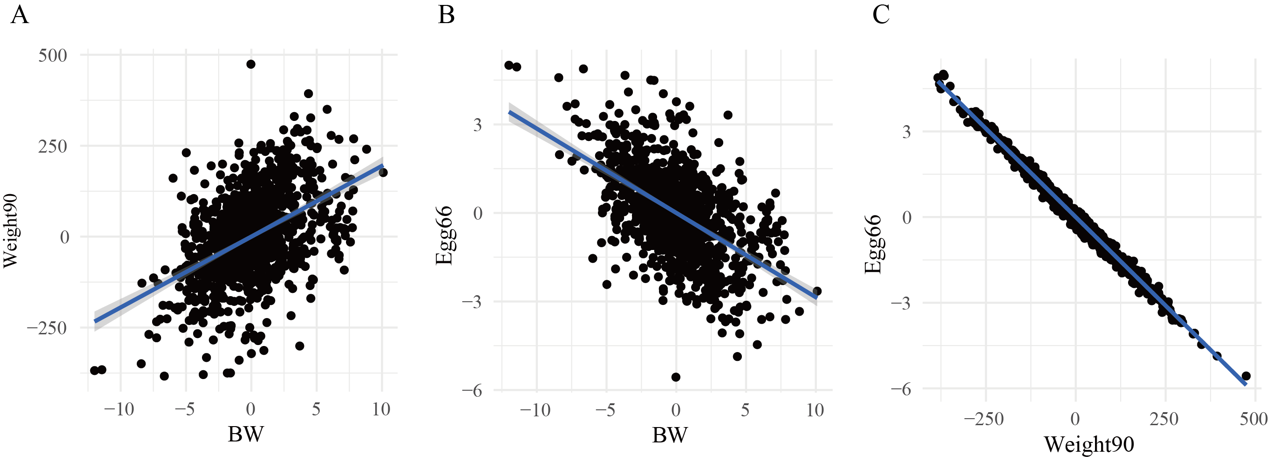


Supplementary Figure S3. Scatter plots showing correlations among estimated breeding values (EBVs) for different traits. Panel A shows the relationship between birth weight (BW; x-axis) and body weight at 90 days (BW90; y-axis). Panel B shows the relationship between birth weight (BW; x-axis) and egg number at 66 weeks (EN66; y-axis). Panel C shows the relationship between body weight at 90 days (BW90; x-axis) and egg number at 66 weeks (EN66; y-axis).


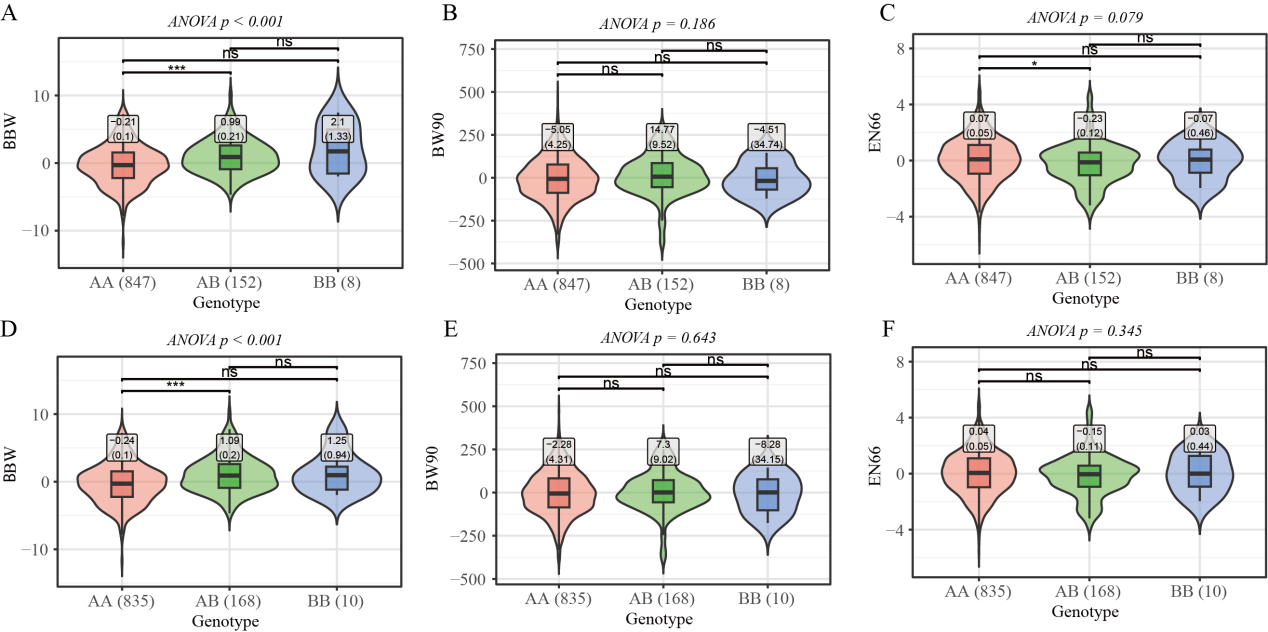


Supplementary Figure S4. Changes in EBVs across genotypes at pleiotropic loci. Panels A-C correspond to CHR25:6006715, and panels D-F correspond to CHR25:6004533. Panels A and D, B and E, and C and F show results for birth weight (BW), body weight at 90 days (BW90), and egg number at 66 weeks (EN66), respectively. The x-axis indicates genotypes (with sample sizes shown in parentheses), and the y-axis represents the EBV of the corresponding trait. Gray boxes denote the mean ± standard error (SE). Statistical significance based on t-tests is indicated above the boxplots.
